# Small RNA Sequencing and Multiplex RT-PCR for Diagnostics of Grapevine Viruses and Virus-like Organisms

**DOI:** 10.3390/v14050921

**Published:** 2022-04-28

**Authors:** Vanja Miljanić, Jernej Jakše, Denis Rusjan, Andreja Škvarč, Nataša Štajner

**Affiliations:** 1 Department of Agronomy, Biotechnical Faculty, University of Ljubljana, 1000 Ljubljana, Slovenia; vanja.miljanic84@gmail.com (V.M.); jernej.jakse@bf.uni-lj.si (J.J.); denis.rusjan@bf.uni-lj.si (D.R.); 2 Chamber of Agriculture and Forestry of Slovenia, Agriculture and Forestry Institute Nova Gorica, 5000 Nova Gorica, Slovenia; andreja.skvarc@go.kgzs.si

**Keywords:** *Vitis vinifera* L., virome, sRNA-seq, mRT-PCR

## Abstract

Metagenomic approaches used for virus diagnostics allow for rapid and accurate detection of all viral pathogens in the plants. In order to investigate the occurrence of viruses and virus-like organisms infecting grapevine from the Ampelographic collection Kromberk in Slovenia, we used Ion Torrent small RNA sequencing (sRNA-seq) and the VirusDetect pipeline to analyze the sRNA-seq data. The used method revealed the presence of: *Grapevine leafroll-associated virus 1* (GLRaV-1), *Grapevine leafroll-associated virus 2* (GLRaV-2), *Grapevine leafroll-associated virus 3* (GLRaV-3), *Grapevine rupestris stem pitting-associated virus* (GRSPaV), *Grapevine fanleaf virus* (GFLV) and its satellite RNA (satGFLV), *Grapevine fleck virus* (GFkV), *Grapevine rupestris vein feathering virus* (GRVFV), *Grapevine Pinot gris virus* (GPGV), *Grapevine satellite virus* (GV-Sat), *Hop stunt viroid* (HSVd), and *Grapevine yellow speckle viroid 1* (GYSVd-1). Multiplex reverse transcription-polymerase chain reaction (mRT-PCR) was developed for validation of sRNA-seq predicted infections, including various combinations of viruses or viroids and satellite RNA. mRT-PCR could further be used for rapid and cost-effective routine molecular diagnosis, including widespread, emerging, and seemingly rare viruses, as well as viroids which testing is usually overlooked.

## 1. Introduction

Grapevine is one of the most susceptible plants to viral infections. More than 86 viruses belonging to different families and genera have been reported to infect grapevine [1], and their number is constantly growing. Recently, two novel members of the genus *Vitivirus* have been identified in South Africa [2].

Most grapevine viruses have an RNA genome, including viruses associated with four major and widespread disease complexes (infectious degeneration and decline, leafroll, rugose wood, and fleck disease complex) [3]. Viruses with a DNA genome have also been identified in grapevine, and they are associated with vein-clearing and vine decline syndrome [4], red blotch disease [5,6], roditis leaf discoloration [7], and fruit tree decline syndrome [8].

Viral pathogens are spread over long distances by infected material (nursery productions), whereas infections within a vineyard or an area are transmitted mechanically and by insects, mites, or nematodes [3]. Viruses and virus-like organisms can cause severe developmental and morphological malformations, affect grapevine physiological activity and metabolism, reduce yield, decrease quality of grapes and wines, and shorten vineyard life, resulting in high economic losses [9,10,11,12]. For example, estimated economic losses caused by *Grapevine leafroll-associated virus 3* (GLRaV-3) in California are more than USD 90 million annually [13]. Therefore, rapid, effective, and reliable detection is crucial to limit their spread.

High-throughput sequencing technology (HTS), which targets all nucleic acid types, enables rapid and accurate detection, including previously described and novel viruses and virus-like organisms [14,15,16,17]. An approach that enables virus discovery through HTS technology and assembly of small RNAs (small RNA sequencing, sRNA-seq) has proven to be highly efficient in the detection of new RNA and DNA grapevine viruses [4,7,18,19], virome studies [20,21,22,23], and to evaluate the efficacy of different elimination methods such as chemotherapy, somatic embryogenesis, and meristem tissue culture [24,25]. All in silico predicted grapevine viral infections are most commonly validated using RT-PCR [21,22,23,24,25,26]. Several other molecular diagnostic methods as well as immunological detection methods, and biological indexing are used in plant virology [27]. However, most routine diagnostic assays can only be used for detection of one target virus/virus-like organism. Multiplex RT-PCR (mRT-PCR)/multiplex PCR (mPCR), which enables simultaneous amplification of several viral entities in a single reaction, is less labor intensive, time saving and cost-effective, especially when a large number of samples needs to be tested for mixed infections. To date, mRT-PCR/mPCR has been used to detect various herbaceous and woody plant-infecting viruses and viroids [28,29,30,31,32,33,34,35,36,37,38,39,40,41,42], including those infecting grapevine [43,44,45,46,47,48,49]. However, mRT-PCR/mPCR has not been used for validation of HTS-predicted viral infections in grapevines thus far.

The aim of the presented work was to perform sRNA-seq for the diagnosis of grapevine viral pathogens in six grapevine varieties from the Ampelographic collection Kromberk, Slovenia, and to develop an mRT-PCR assay for the validation of sRNA-seq data that could be further used for rapid and cost-effective routine molecular diagnosis in large-scale surveys.

## 2. Materials and Methods

### 2.1. Plant Material

**Figure 1:**
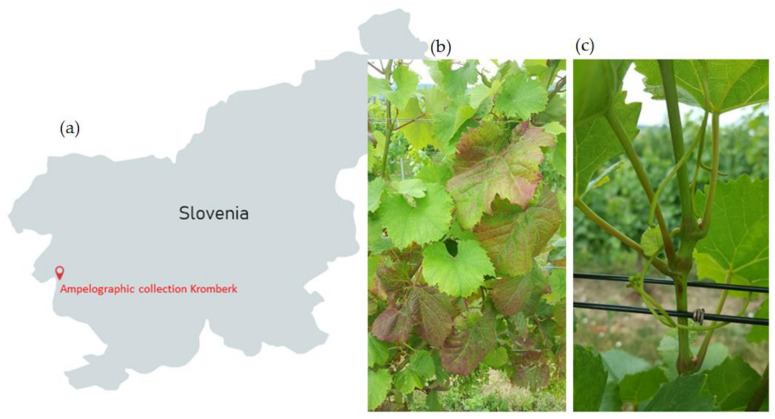
(**a**) Location of the Ampelographic collection Kromberk 45°57′40.8″ N 13°39′44.7″ E; (**b**) redding of the interveinal areas caused by GLRaV-3 on the ‘Pokalca’ variety; (**c**) shoot malformation (shorten internodes) caused by GFLV on the ‘Rebula’ variety.

A total of 13 cuttings from six grapevine varieties, two red, ‘Cipro’ (‘Rosenmuscateller’) and ‘Pokalca’ (‘Schioppettino’), and four white, ‘Malvazija’ (‘Malvasia d’Istria’), ‘Volovnik’ (‘Vela pergola’), ‘Rebula’ (‘Ribolla gialla’), and ‘Poljšakica’, were collected from the Ampelographic collection Kromberk near Nova Gorica, Slovenia, in 2017 (Figure 1). Cuttings were sprouted in water at room temperature (21 °C) at the Biotechnical Faculty, University of Ljubljana. Developed young leaves were sampled and stored at –80 °C for further analysis.

### 2.2. Small RNA Isolation, Library Construction, sRNA-Seq and Bioinformatics Analysis

The selected samples were pooled together into four pools representing either samples of the same variety (L1, L2, and L3) or of different varieties (L4). Small RNAs (sRNAs) were isolated using mirVana™ miRNA Isolation Kit (Ambion, Life Technologies, Waltham, MA, USA) according to the manufacturer’s instructions for the enrichment of sRNAs. The quantity and quality of sRNAs were assessed using the Agilent 2100 Bioanalyzer (Agilent Technologies, Inc., Santa Clara, CA, USA) according to the manufacturer’s instructions. Libraries of sRNAs were constructed using the Ion Total RNA-Seq Kit v2 (Ion Torrent™, Waltham, MA, USA) and were barcoded using the Xpress™ RNA-Seq Barcode 1–16 Kit (Ion Torrent™, Waltham, MA, USA) according to the manufacturer’s instructions. The yield and size distribution of the amplified cDNA libraries were determined using the Agilent 2100 Bioanalyzer (Agilent Technologies, Inc., Santa Clara, CA, USA). Libraries were pooled at equimolar concentrations and prepared for sequencing using the Ion PI™ Hi-Q™ OT2 200 Kit and Ion PI™ Hi-Q™ Sequencing 200 Kit (Ion Torrent™, Waltham, MA, USA) according to the manufacturer’s instructions. Sequencing was performed on Ion PI™ chips v3 using an Ion Proton™ System (Ion Torrent™, Waltham, MA, USA), according to the manufacturer’s instructions. Raw sequencing data were deposited in the National Center for Biotechnology Information (NCBI) Sequence Read Archive (SRA) database under BioProject number PRJNA667593, BioSamples: SAMN16378719-SAMN16378722. The sRNA-seq data were analyzed using the VirusDetect pipeline with default parameters [50]. The pipeline performs reference-guided assembly using the Burrows–Wheeler Aligner (BWA) and de novo assembly using the Velvet Genomic Assembler. The plant virus database was used as reference, and the grapevine genome was selected to subtract host sRNAs.

### 2.3. mRT-PCR for Validation of sRNA-Seq Predicted Viral Pathogens

Confirmation of sRNA-seq-predicted infections was performed by mRT-PCR. Total RNA was extracted from 100 mg of frozen leaves using the RNeasy Plant Mini Kit (Qiagen, Hilden, Germany). First strand cDNA synthesis was performed using the High-Capacity cDNA Reverse Transcription Kit (Applied Biosystems™, Foster City, CA, USA) according to the manufacturer’s instructions. mRT-PCR was performed using the KAPA2G Fast Multiplex PCR Kit (KAPA Biosystems, Wilmington, MA, USA). The reaction mixture was prepared using 12.5 µL of KAPA2G Fast Multiplex Mix (KAPA2G Fast HotStart DNA Polymerase, KAPA2G Buffer A, 0.2 mM of each dNTP, 3 mM MgCl_2_, and stabilizers), 0.2 µL (0.1 µL for GRVFV) of each 10 µM forward and reverse primer (final concentration 0.08 µM; for GRVFV 0.04 µM), 1 µL of pooled cDNA, and nuclease-free water up to 25 µL. Primers are listed in Table 1. Amplification was performed in a thermal cycler (Applied Biosystems™, Waltham, MA, USA) under the following conditions: initial denaturation at 95 °C for 3 min, 35 cycles consisting of a denaturation step at 95 °C for 15 s, annealing at 58 °C for 30 s, extension at 72 °C for 1 min, and a final extension at 72 °C for 1 min. The amplified products were analyzed by electrophoresis on 1.2% agarose gel, stained with ethidium bromide, and visualized under a UV transilluminator. Amplicons sizes were determined by comparison with the GeneRuler™ 100 bp Plus DNA Ladder (Thermo Fisher Scientific, Waltham, MA, USA).

**Table 1:** List of primers used for mRT-PCR detection.

Viral Pathogen	Primer Name	Primer Sequence (5′-3′)	Product Size *	Tm *	GC % *	Amplified Region	Reference
GLRaV-3	LR3-8504V	ATGGCATTTGAACTGAAATT	942 bp	51.81	30.00	CP	[51]
	LR3-9445C	CTACTTCTTTTGCAATAGTT		48.91	30.00		
GLRaV-2	LRaV-2 (1)	AGGCGGATCGACGAATAC	821 bp	56.64	55.56	hsp70-like protein, p63	[52]
	LRaV-2 (2)	ATCCTGTCCGGCGCTGTG		62.46	66.67		
GPGV	Pg-Mer-F1	GGAGTTGCCTTCGTTTACGA	770 bp	58.21	50.00	MP/CP	[53]
	Pg-Mer-R1	GTACTTGATTCGCCTCGCTCA		60.47	52.38		
GRVFV	GRVFV_6090F	CATCGTTCTGATCCTCAGCC	516 bp	58.14	55.00	polyprotein	[54]
	GRVFV_6605R	AGAGACGCTGACCATGCCAC		62.51	60.00		
GFLV	GFLV_13_16_F	TGACACGTGCCTTTATTGGA	488 bp	57.45	45.00	polyprotein, segment RNA2	[23]
	GFLV_13_16_R	CTCAAGTTGGGGAAGGTCAA		57.34	50.00		
GLRaV-1	CPd2/F	GTTACGGCCCTTTGTTTATTATGG	398 bp	58.42	41.67	CPd2	[55]
	CPd2/R	CGACCCCTTTATTGTTTGAGTATG		57.88	41.67		
GRSPaV	RSP 48	AGCTGGGATTATAAGGGAGGT	330 bp	57.63	47.62	CP	[56]
	RSP 49	CCAGCCGTTCCACCACTAAT		60.04	55.00		
GV-Sat	GV-Sat_for	CCCGGACTCACATTAAGTCAA	305 bp	57.67	47.62	ORF1, ORF2, 3′UTR	[57]
	GV-Sat_rev	GCACAAGCGAGATAACAGCA		58.92	50.00		
GFkV	GFkVf	TGACCAGCCTGCTGTCTCTA	179 bp	60.25	55.00	CP	[44]
	GFkVr	TGGACAGGGAGGTGTAGGAG		59.96	60.00		
satGFLV	FP3-F	GTGGSCCCGCRAGTGT	870 bp	degenerative primer pair		hypothetical protein	[58]
	RP-R	TAAWGAGCAACCAAAATCCCA					
HSVd	HSV-78P	AACCCGGGGCAACTCTTCTC	~300 bp	62.13	60.00	complete genome	[59]
	HSV-83M	AACCCGGGGCTCCTTTCTCA		63.34	60.00		
GYSVd-1	-	TGTGGTTCCTGTGGTTTCAC	~368 bp	58.24	50.00	complete genome	[60]
	-	ACCACAAGCAAGAAGATCCG		58.19	50.00		

* Determined with Primer-BLAST.

## 3. Results

### 3.1. Viruses and Virus-like Organisms Detected by sRNA-Seq

sRNA-seq of pooled grapevine samples resulted in 17,195,263–18,713,942 reads per pool (Table 2). Of the total reads, 50.12–67.22% were mapped to the grapevine genome, while 3.06–11.98% were mapped to viral genomes (Table 2). After concatenating unique reference-guided contigs and unique de novo assembled contigs and after removing redundancies, 461–1102 unique viral contigs were generated (Table 2). The total number of reference viral sequences identified by BLASTN search per library is presented in Table 2, while for each viral pathogen, it is presented in Table S1.

**Figure 2:**
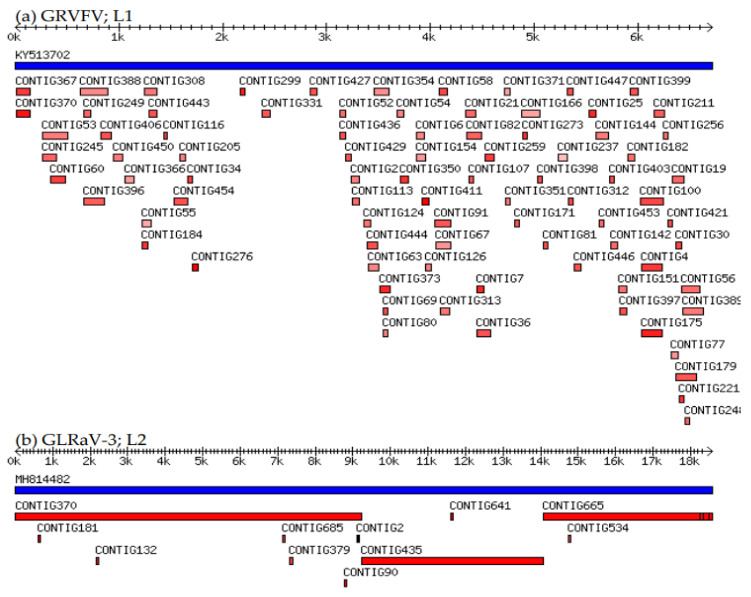
Virus-assembled contigs (red bars) mapped to complete reference genome sequence (blue bars): (**a**) GRVFV, virus with the lowest reference genome coverage (61.99%; L1); (**b**) GLRaV-3, virus with the highest reference genome coverage (99.96%; L2).

Out of identified references, we selected one complete genome sequence per viral pathogen in each library that had the highest coverage. The used method revealed the presence of: *Grapevine leafroll-associated virus 1* (GLRaV-1), *Grapevine leafroll-associated virus 2* (GLRaV-2), *Grapevine leafroll-associated virus 3* (GLRaV-3), *Grapevine rupestris stem pitting-associated virus* (GRSPaV), *Grapevine fanleaf virus* (GFLV) and its satellite RNA (satGFLV), *Grapevine fleck virus* (GFkV), *Grapevine rupestris vein feathering virus* (GRVFV), *Grapevine Pinot gris virus* (GPGV), *Grapevine satellite virus* (GV-Sat), *Hop stunt viroid* (HSVd) and *Grapevine yellow speckle viroid 1* (GYSVd-1). The highest number of viral entities (nine) was found in the library that was a mixture of three different varieties (L4). Eight viral pathogens were detected in the library of variety ‘Cipro’ (L1), while in the other two libraries (L2 and L3), the number of identified viral pathogens was seven (Table 3). The coverage with references from the database was between 61.99% (GRVFV, L1) and 99.96% (GLRaV-3, L2) (Table 3; Figure 2), with a sequencing depth between 7X (GRSPaV, L1) and 5313.2 X (satGFLV, L2) (Table 3). GLRaV-1, GLRaV-2, and GV-Sat were present only in one library (L1). GFkV was detected in L3 and L4, and GFLV and satGFLV were detected in L2 and L4. GFLV possesses a bipartite genome; thus, the sRNA-seq data for RNA1 and RNA2 are shown in Table 3. GLRaV-3 was detected in three libraries (L2, L3, and L4) and had the highest coverage (99.80–99.96%) among viruses in all three libraries. GRSPaV, GPGV, and GRVFV were detected in all libraries. GRSPaV had low sequencing depth in all libraries (7X, 8.9X, 10.4X, and 9.7X, respectively). GRVFV had the lowest references coverage in all libraries (61.99%, 70.24%, 68.47%, and 70.21%, respectively). Considering viroids, HSVd was detected in all libraries, while GYSVd-1 was absent only in L2 (Table 3).

### 3.2. mRT-PCR for Validation of sRNA-Seq Predicted Viral Pathogens

**Figure 3:**
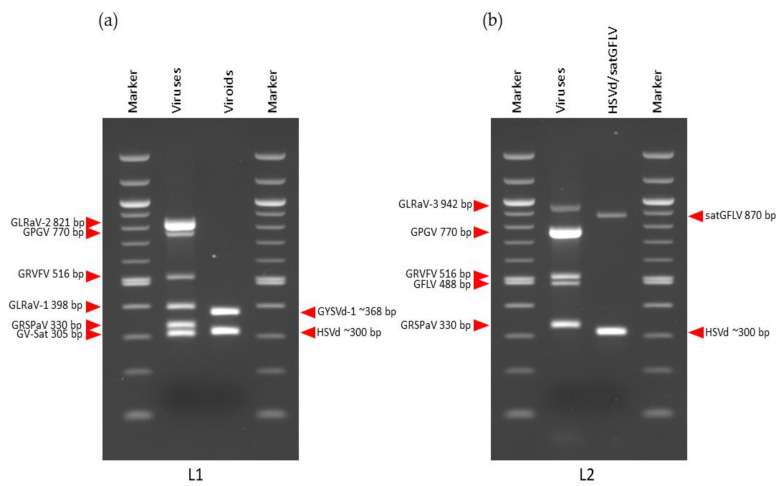

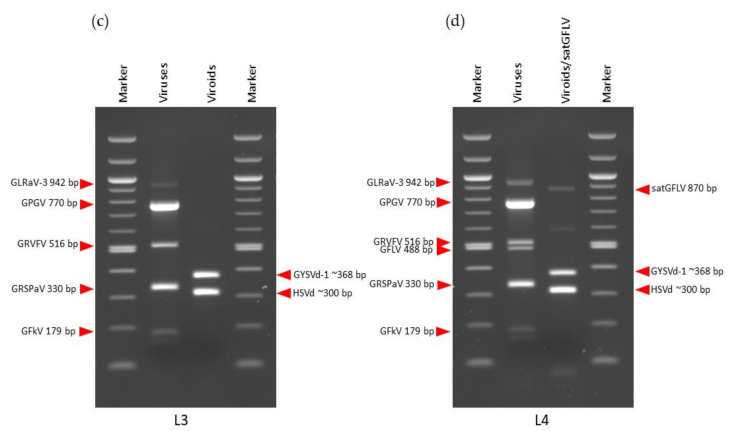
Validation of sRNA-seq-predicted viruses and virus-like organisms with mRT-PCR: (**a**) L1; (**b**) L2; (**c**) L3; (**d**) L4.

Primer combinations with different expected amplified fragments were chosen for mRT-PCR to allow for differentiation on the agarose gel. All primers corresponded to those found in the literature (Table 1). The primers for GV-Sat and GFLV had been designed in our previous studies [23,57]. Several parameters such as primer concentration (0.04–0.2 µM), annealing temperature (55–60 °C), number of cycles (30–35), and amount of cDNA (1 µL and 2 µL) were optimized to determine the best conditions for simultaneous amplification of the predicted infections. As under-amplified amplicons were obtained with a higher primers concentration (0.2 µM), it was reduced to 0.08 µM. With this primers concentration (0.08 µM) and an annealing temperature of 55 °C, all predicted viruses were amplified in all libraries, although nonspecific banding patterns of approximately 250 bp were also observed. In an effort to reduce these background bands, the annealing temperature was increased to 58 °C, and the concentration of the primer pair (GRVFV_6090F/GRVFV_6605R) amplifying 516 bp of GRVFV polyprotein product (Table 1) was decreased to 0.04 µM. Better results were obtained with a lower amount of cDNA (1 µL), compared with 2 µL (data not shown). Under these conditions (primer concentration 0.08 µM and 0.04 µM for GRVFV, annealing temperature 58 °C, 35 cycles and 1 µL of cDNA), specific RT-PCR amplification products of the expected sizes were obtained for all viral pathogens in all libraries. Different combinations of viruses were amplified simultaneously in all four libraries: L1 (GV-Sat, GRSPaV, GLRaV-1, GRVFV, GPGV, and GLRaV-2); L2 (GRSPaV, GFLV, GRVFV, GPGV, GLRaV-3); L3 (GFkV, GRSPaV, GRVFV, GPGV, GLRaV-3); L4 (GFkV, GRSPaV, GFLV, GRVFV, GPGV, GLRaV-3) (Figure 3). In addition, different combinations of viroids/satGFLV were amplified simultaneously: L1 and L3 (HSVd, GYSVd-1), L2 (HSVd, satGFLV), L4 (HSVd, GYSVd-1, satGFLV) (Figure 3).

## 4. Discussion

Thirteen grapevines of six important autochthonous and local varieties were screened for viruses and virus-like organisms with sRNA-seq. A total of 70,902,637 reads were generated, and 5,272,381 (7.44%) were mapped to viral reference sequences, while 42,342,716 (59.72%) originated from grapevine. The BLASTN search of the unique viral-assembled contigs revealed the presence of widespread viruses associated with four major disease complexes, emerging virus, GV-Sat (first report in Slovenia) [57], as well as worldwide-distributed viroids. A high number of contigs and their short length were observed for GRSPaV, GFLV, GRVFV and GFkV, which is in accordance with our previous study [23], and may be related with their high genetic variability. For example, GFLV (RNA1) reference sequence (JX513889), which is 7340 nt long, was covered with 127 contigs (Figure S1). In contrast, GLRaV-2 reference sequence (FJ436234), which is 16,486 nt long, was covered with only eight contigs, from which one was long enough to cover 99.86% of the references (Figure S2).

Additionally, in this study, we described the application of the mRT-PCR approach for validation of the sRNA-seq data. Simultaneous amplifications of different combinations of nine viruses or two viroids and satGFLV were performed. According to the KAPA2G Fast Multiplex Kit protocol, employed primers should have a similar temperature melting (Tm) and GC content of 40–60%. In our study, the Tm of primers used for virus amplification was not similar; the lowest Tm had a primer pair for GLRaV-3 amplification (51.81 °C for forward and 48.91 °C for reverse primer) (Table 1). Considering GC content, according to the protocol primers, a GC content higher than 60% may require higher and/or longer denaturation temperature and time, while a GC content lower than 40% may require increased primer concentrations, additional MgCl_2_ and/or annealing temperature lower than 60 °C (KAPA2G Fast Multiplex PCR Kit, https://www.n-genetics.com/products/1104/1023/12664.pdf, accessed on 1 April 2022). In this study, the lowest GC content had again primers for GLRaV-3 amplification (30%), while all other primers for amplification of predicted viruses in L2, L3, and L4 had GC content in the range of 40–60%. In L1, all primers for virus amplification had a GC content of 40–60%, except for the reverse primer of GLRaV-2 (66.67%) (Table 1). Although the primers in our study had differences in Tm and GC content in all cases, successful amplifications were obtained (Figure 3). Thus far, the highest number of grapevine viral pathogens amplified using mRT-PCR was nine (ArMV, GFLV, GVA, GVB, GRSPaV, GFkV, GLRaV-1, GLRaV-2, and GLRaV-3) [44,47]. Nassuth et al. (2000) [43] reported simultaneous detection of ArMV, GRSPaV, and malate dehydrogenase mRNA for GLRaV-3, GVA, GVB and RubiscoL mRNA. Simultaneous detection of grapevine-infecting viruses belonging to the *Nepovirus* genus were reported by Digiaro et al. (2007) [45]. Hajizadeh et al. (2012) [46] developed mRT-PCR for simultaneous detection of five grapevine viroids. Simultaneous amplification of viruses and viroids have also been reported: GFLV, GYSVd-1, and GYSVd-2, in addition, HSVd was included instead of plant internal control [48], and for GPGV, GFkV, HSVd and GYSVd-1 [49]. In this study, a cumulative number of viral pathogens was minimum 7 and maximum 9 per library. Considering that viroids may form dimers or even multimers that are also visible on agarose gel, the viroids were separately amplified. Some studies found that mRT-PCR is less sensitive compared to singleplex RT-PCR, which specifically targets one viral pathogen [61,62]. Lower detection sensitivity has also been reported when more than five primer pairs were used in a single reaction to detect stone fruit viruses [36].

However, in this study, we showed that mRT-PCR is highly effective, reliable, and sensitive, enabling validation of all viral pathogens predicted with sRNA-seq.

High-throughput screening and high-throughput validation of viral entities for some important old grapevine varieties from the Ampelographic collection Kromberk, Slovenia, was performed. The mRT-PCR protocol described herein provides a simple, time-saving, cost-efficient method for the rapid and reliable validation of sRNA-seq data and successful detection of viral pathogens belonging to different families and genera.

## Figures and Tables

**Table 2:** Summary of results obtained with VirusDetect.

Library Label	Samples	BioSample ID	Total No. of Reads	Viral Mapping	Grapevine Mapping	Final Unique Viral Contigs	References Identified by BLASTN Search
L1	3 ‘Cipro’	SAMN16378719	17,398,590	1,835,271 (10.55%)	10,437,476 (59.99%)	461	62
L2	3 ‘Malvazija’	SAMN16378720	17,594,842	2,108,476 (11.98%)	8,818,062 (50.12%)	699	128
L3	3 ‘Volovnik’	SAMN16378722	18,713,942	571,865 (3.06%)	12,578,899 (67.22%)	882	98
L4	2 ‘Rebula’ 1 ‘Pokalca’ 1 ‘Poljšakica’	SAMN16378721	17,195,263	756,769 (4.40%)	10,508,279 (61.11%)	1102	203

**Table 3:** Viruses and virus-like organisms detected with BLASTN search (VirusDetect pipeline).

Library Label	Detected Viral Pathogens	Reference Sequence	Reference Origin	Reference Length	Consensus Length	Reference Coverage (%)	No. of Contigs	Sequencing Depth	Nucleotide Identity (%)
L1	GLRaV-1	MG925332	France	18,863	18,608	98.65	36	343.8	94.22
	GLRaV-2	FJ436234	USA	16,486	16,463	99.86	8	1254.3	99.43
	GRSPaV	KX035004	France	8743	6058	69.29	64	7	95.84
	GPGV	KP693444	Czech Republic	7172	7089	98.84	12	586.3	95.96
	GRVFV	KY513702	Switzerland	6716	4163	61.99	85	21.4	92.6
	GV-Sat	KC149510	USA	1060	969	91.42	6	1567	95.74
	HSVd	KJ810551	Taiwan	309	309	100	4	1257.4	93.93
	GYSVd-1	KP010010	Thailand	389	389	100	4	1951.3	96.92
L2	GLRaV-3	MH814482	unknown	18,580	18,572	99.96	11	142.3	99.55
	GRSPaV	KX035004	France	8743	7978	91.25	52	8.9	98.05
	GPGV	MN458445	France	7269	7254	99.79	4	134.1	97.63
	GFLV (RNA1)	JX513889	Canada	7340	7302	99.48	127	897.8	90.47
	GFLV (RNA2)	MN496418	France	3743	3521	94.07	55	2738	90.77
	satGFLV	KR014543	Slovenia	989	933	94.34	13	5313.2	92.96
	GRVFV	MF000326	New Zealand	6701	4707	70.24	62	224	87.97
	HSVd	KY508372	Mexico	316	314	99.37	5	1645.3	93.26
L3	GLRaV-3	MH814485	unknown	18,656	18,618	99.8	8	253.9	98.5
	GRSPaV	JQ922417	USA	8758	8462	96.62	60	10.4	96.28
	GPGV	MN458445	France	7269	7254	99.79	5	114.4	96.79
	GFkV	AJ309022	Italy	7564	6654	87.97	83	113.8	94.28
	GRVFV	KY513701	France	6730	4608	68.47	111	50.1	90.54
	HSVd	KJ810551	Taiwan	309	309	100	3	1233.8	95.19
	GYSVd-1	KP010010	Thailand	389	389	100	2	1931.4	97.62
L4	GLRaV-3	MH814482	unknown	18,580	18,565	99.92	19	45.3	99.46
	GRSPaV	KX035004	France	8743	8427	96.39	67	9.7	96.4
	GPGV	MN458445	France	7269	7257	99.83	11	189.7	97.43
	GFLV (RNA1)	KX034843	France	7347	6957	94.69	100	411.7	89.95
	GFLV (RNA2)	MG418840	France	3777	3517	93.12	54	989.7	91.03
	satGFLV	KR014587	Slovenia	863	617	71.49	4	21.8	97.64
	GFkV	AJ309022	Italy	7564	6454	85.33	47	82.2	95.67
	GRVFV	KY513702	Switzerland	6716	4715	70.21	125	37.3	93.05
	HSVd	KJ810551	Taiwan	309	309	100	4	2036.3	94.34
	GYSVd-1	MF510389	Hungary	368	368	100	3	942.2	97.46

## Data Availability

The datasets generated and analyzed during the current study are available in the NCBI Sequence Read Archive (SRA) repository (https://www.ncbi.nlm.nih.gov/sra/, accessed on 1 April 2022) under the BioProject accession number PRJNA667593.

## Supplementary Materials

The following supporting information can be downloaded at: https://www.mdpi.com/article/10.3390/v14050921/s1. Table S1: Number of reference viral sequences identified by BLASTN search (VirusDetect pipeline); Figure S1: GFLV (RNA1)-assembled contigs (red bars) mapped to reference sequence (JX513889) (blue bars); Figure S2: GLRaV-2-assembled contigs (red bars) mapped to reference sequence (FJ436234) (blue bars).
