# Supplementary material for: Small RNA Sequencing and Multiplex RT-PCR for Diagnostics of Grapevine Viruses and Virus-like Organisms
Source: Viruses. 2022 Apr 28;14(5):921. doi: 10.3390/v14050921 (PMC9145883; doi:10.3390/v14050921)
Supplement: Supplementary file 1 [file viruses-14-00921-s001.zip › viruses-1688529-supplementary.pdf]

viruses

MDPI

Supplementary Material

Small RNA Sequencing and Multiplex RT-PCR for Diagnostics of Grapevine Viruses and Virus-Like Organisms

Table S1. Number of reference viral sequences identified by BLASTN search (VirusDetect pipeline).

Library label	GLRaV-1	GLRaV-2	GLRaV-3	GRSPaV	GFLV	GFkV	GRVFV	GPGV	GV-Sat	satGFLV	HSVd	GYSVd-1
**L1**	3	1	/	23	/	/	31	1	1	/	1	1
**L2**	/	/	1	21	81	/	19	1	/	4	1	/
**L3**	/	/	1	36	/	11	46	2	/	/	1	1
**L4**	/	/	1	46	103	3	42	2	/	4	1	1

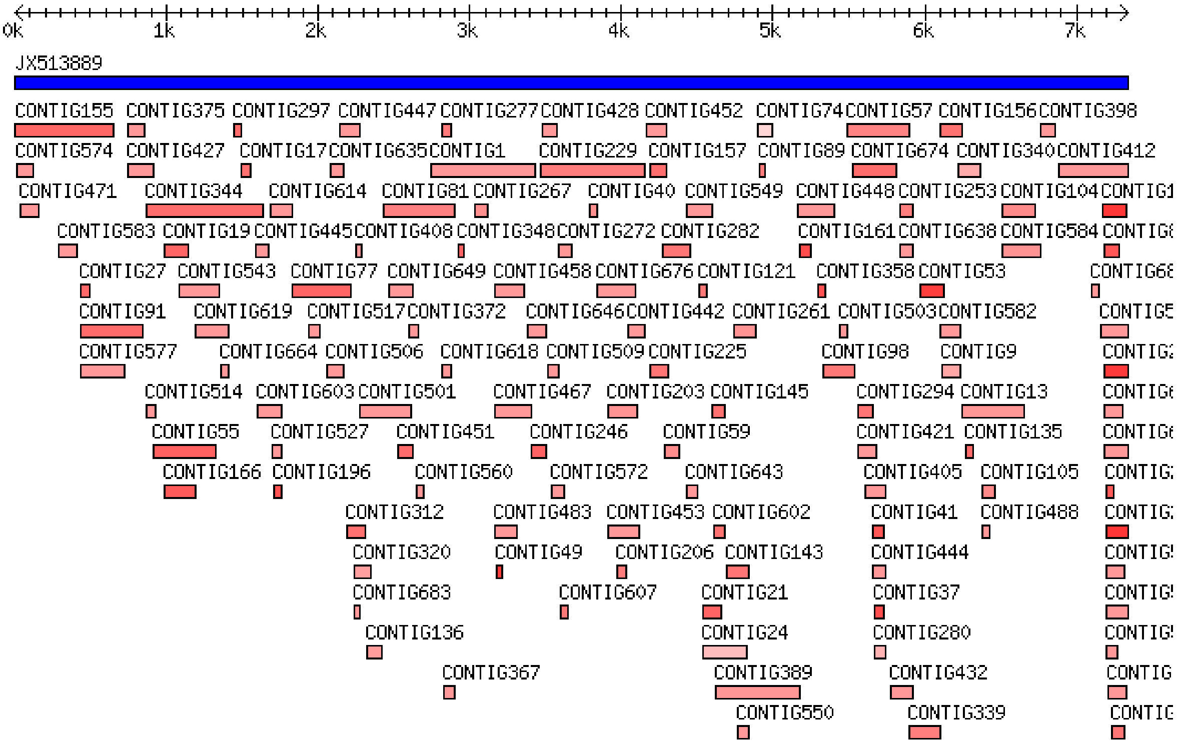

Figure S1. GFLV (RNA1) assembled contigs (red bars) mapped to reference sequence (JX513889) (blue bars). Reference was covered with 127 contigs.

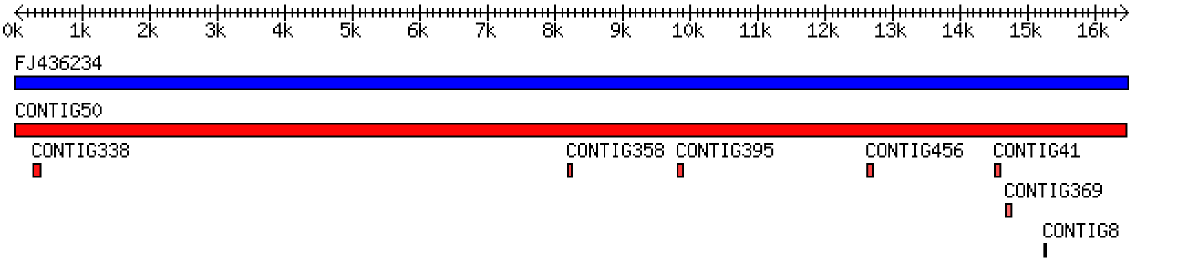

Figure S2. GLRaV-2 assembled contigs (red bars) mapped to reference sequence (FJ436234) (blue bars). Reference was covered with 8 contigs, but just one was enough to cover 99.86% of reference genome.
